# Vascular endothelial growth factor, endostatin levels and clinical features among patients with ulcerative colitis and irritable bowel syndrome and among healthy controls: a cross-sectional analytical study

**DOI:** 10.1590/1516-3180.2018.0274161118

**Published:** 2018-12-13

**Authors:** Evrim Kahramanoğlu Aksoy, Hülya Çetinkaya, Berna Savaş, Arzu Ensari, Murat Torgutalp, Cumali Efe

**Affiliations:** I MD. Physician, Department of Gastroenterology, Keçiören Training and Research Hospital, Ankara, Turkey.; II MD. Professor, Department of Gastroenterology, Ankara University Faculty of Medicine, Ankara, Turkey.; III MD. Professor, Department of Pathology, Ankara University Faculty of Medicine, Ankara, Turkey; IV MD. Professor, Department of Pathology, Ankara University Faculty of Medicine, Ankara, Turkey.; V MD. Physician, Department of Rheumatology, Ankara University Faculty of Medicine, Ankara, Turkey.; VI MD. Associate Professor, Department of Gastroenterology, Gazi Yaşargil Training And Research Hospital, Diyarbakır, Turkey.

**Keywords:** ulcerative, Vascular endothelial growth factors, Endostatins

## Abstract

**BACKGROUND:**

Increased angiogenetic activity in inflammatory bowel disease (IBD) has been shown in previous studies. The aim of this study was to evaluate the relationship of serum vascular endothelial growth factor (VEGF) and endostatin levels with clinical features and mucosal expression in patients with ulcerative colitis (UC).

**DESIGN AND SETTING:**

Cross-sectional analytical study conducted in a tertiary-level public hospital.

**METHODS:**

Serum VEGF and endostatin levels were determined in 82 individuals: 39 with UC, 28 with irritable bowel syndrome (IBS) and 15 healthy controls (HCs), using enzyme-linked immunosorbent assays (ELISA). VEGF and endostatin expressions were studied using immunohistochemistry (IHC).

**RESULTS:**

Mean serum VEGF and endostatin levels were significantly higher in patients with UC than in patients with IBS and in HCs (511.9 ± 377.5 pg/ml, 305.0 ± 121.42 pg/ml and 36.1 ± 40.6 pg/ml; P = 0.001 for VEGF; and 155.50 ± 59.8 ng/ml, 116.9 ± 23.8 ng/ml and 102.2 ± 22.4 ng/ml; P < 0.001 for endostatin, respectively). There was a positive correlation between serum VEGF and endostatin levels (*r* = 0.422; P < 0.01). Mean H-scores for VEGF expression were higher in the active UC group than in the inactive UC and IBS groups, in the stroma, endothelium and epithelium. Mean H-scores for endostatin expression were higher in the active UC group than in the inactive UC and IBS groups, in the stroma and endothelium. There was no endostatin expression in the epithelium.

**CONCLUSION:**

Increased endostatin appears to be a defensive reaction to increased VEGF in patients with UC.

## INTRODUCTION

Ulcerative colitis is characterized by chronic inflammation and ulceration of the colonic mucosa. Several environmental and genetic factors are responsible for chronic inflammation.^1^ Angiogenesis has been defined as growing of new blood vessels from the preexisting ones. In addition to playing a role in physiological events such as wound healing and growth, it is also seen in tumor development, metastasis, and chronic inflammation.^2^

In inflammatory bowel disease (IBD), although angiogenesis is necessary for ulcer healing and tissue regeneration through providing oxygen and nutrients to the healing zone, it turns into a pathological process through inflow of inflammatory cells and cytokines. Under the influence of inflammatory mediators such as cytokines, growth factors and proteases that come to the healing site, angiogenesis increases further and the microvascular bed enlarges. In this manner, this condition becomes a vicious circle that attracts more inflammatory mediators to the center. It thus leads to chronic inflammation.^3^^,^^4^

Previous studies have shown the existence of several angiogenic factors that may become elevated, such as vascular endothelial growth factor (VEGF), basic fibroblast growth factor (bFGF), platelet derived growth factor (PDGF), angiogenin and angiopoietin-2.^5^^,^^6^^,^^7^^,^^8^ VEGF is defined as the potent proangiogenic factor that is secreted by parenchymal, endothelial and activated immune cells.^9^ Elevated serum and tissue levels of VEGF in patients with IBD have been shown in many studies.^5^^,^^6^^,^^7^^,^^9^^,^^10^ Kanazawa et al. and Kapsoritakis et al. found elevated serum VEGF-A levels in IBD patients with active disease. Griga et al. showed that there was increased expression of VEGF in the inflamed intestinal mucosa of patients with active IBD.^5^^,^^6^^,^^11^ Chidlow et al. and Ardelean et al. demonstrated the effect of anti-angiogenic treatment through decreased angiogenic and histopathological activity in the inflamed mucosa.^3^^,^^4^^,^^12^

Endostatin is a 20-kDa fragment of collagen-18 that is generated by proteinases such as matrix metalloproteinases (MMPs), especially MMP-9. It acts as an endogenous angiogenesis inhibitor through inhibiting proliferation and inducing apoptosis of endothelial cells.^13^ Endostatin downregulates many angiogenic factors, such as VEGF, bFGF, hepatocyte growth factor (HGF), hypoxia-induced-factor-1α (HIF-1α) and tumor necrosis factor-α (TNF-α) and it upregulates anti-angiogenic genes such as thrombospondin-1, HIF-1α-inhibitor, etc.^14^

Studies on the effects of endostatin in cases of ulcerative colitis have produced contradictory results. Sandor et al. reported the presence of increased levels of endostatin and angiostatin in the colonic mucosa, rather than increased levels of VEGF, in a study on experimental colitis. They speculated that the reason for the chronicity of the disease, decreased healing of mucosal lesions and lack of increased VEGF levels was the increased levels of endostatin and angiostatin.^15^ In another study, they reported that the effect of 5-aminosalicylate acid (5-ASA) on the healing of ulcerative colitis (UC) may be related to downregulation of the anti-angiogenic factors endostatin and angiostatin.^16^

In the present study, we aimed to evaluate the relationship between serum VEGF and endostatin levels and the clinical features of patients with ulcerative colitis, and to evaluate VEGF and endostatin expression in the colonic mucosa of these patients.

## METHODS

### Participants

The participants of this study were recruited according to their consecutive admittance to a gastroenterology outpatient clinic: 39 UC patients, 28 patients with irritable bowel syndrome (IBS) and 15 healthy controls (HCs) who came for consultations between September 2007 and July 2008 were included in this study. The sample size was calculated with a 5% error margin and 80% power, in accordance with the prescriptions of Liou et al.,^17^ yielding an ideal sample of 24 participants for the study group (21 for studying VEGF and 13 for studying endostatin) and 15 for the control group.

The UC clinical activity index (UCAI) was calculated as described by Seo et al.^18^ The Rachmilewitz endoscopic activity index (EAI) was also calculated for the UC group.^19^ All of the IBS patients met the Rome III criteria for their diagnosis. HCs were selected from among individuals who had been admitted because of dyspeptic symptoms and who had normal endoscopic examinations and laboratory tests. The IBS patients and HCs had normal erythrocyte sedimentation rate (ESR) and C-reactive protein (CRP) levels and no parasitic infections in their stool tests. All the patients were between 18 and 65 years of age.

Patients who presented the following conditions were excluded: chronic kidney disease; chronic liver disease; oncological diseases; pregnancy or breastfeeding; use of nonsteroidal anti-inflammatory drugs, anticoagulants or antithrombotic drugs; or previous abdominal surgery.

**Figure 1.**
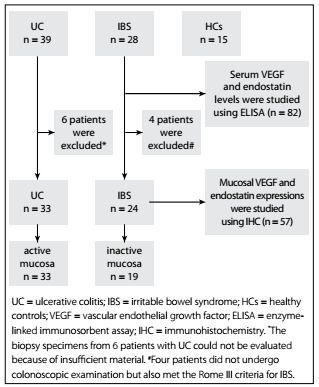
Flowchart of the study.

Serum VEGF and endostatin levels were measured in all groups. All UC patients and 24 IBS patients underwent colonoscopic examination, and colonic biopsy samples were taken from all of these patients.Four patients who did not undergo colonoscopic examination also met the Rome III criteria for IBS. They had normal ESR and CRP levels and no parasitic infection in their stool tests, and they did not want to undergo colonoscopy.The biopsy specimens from six patients with UC could not be evaluated because of insufficient material. VEGF and endostatin expressions were studied by means of immunohistochemical analysis using 33 active mucosal samples and 19 inactive mucosal samples from 33 patients with UC, and using 24 normal mucosal samples from 24 patients with IBS (Figure 1).

This study was approved by the Medical Ethics Committee of Ankara University Faculty of Medicine (June 16, 2008; approval no. 132-3784) and was conducted in accordance with the revised Helsinki Declaration. Informed consent was obtained from all patients.

### Serum VEGF and endostatin levels

Venous blood samples were taken after overnight fasting and were centrifuged at 10,000 rpm for 10 minutes. The resultant serum was collected and kept at -80 °C until the examination date. Serum VEGF levels and endostatin levels were measured in duplicate by means of enzyme-linked immunosorbent assays (ELISA) using commercial kits in accordance with the manufacturers’ instructions (for VEGF: Biosource International, California, USA; and for endostatin: R&D Systems, Minneapolis, MN, USA).

### Immunohistochemical analysis

Paraffin-embedded tissue material from the patients with UC (n = 33) and IBS (n = 24) was retrieved from the pathology archives. Tissue sections of 4 µm were cut and mounted on poly-L-lysin coated slides. The sections were deparaffinized and rehydrated through a graded ethanol series and were then incubated in methanol containing 0.3% H_2_O_2_ to inhibit endogenous peroxidase. Immunostaining was performed using the avidin-biotin-peroxidase complex technique, using 3,3’-diaminobenzidine as the chromogen.

The primary antibodies that were used were recombinant human VEGF (Thermo Fisher Scientific, Fremont, CA, USA; 1/100 dilution) and human monoclonal endostatin (Hycult Biotechnology, Netherlands; 1/15 dilution). Colonic mucosal tissue was used as the positive control for VEGF and prostate tissue was used as the positive control for endostatin.

**Figure 2.**
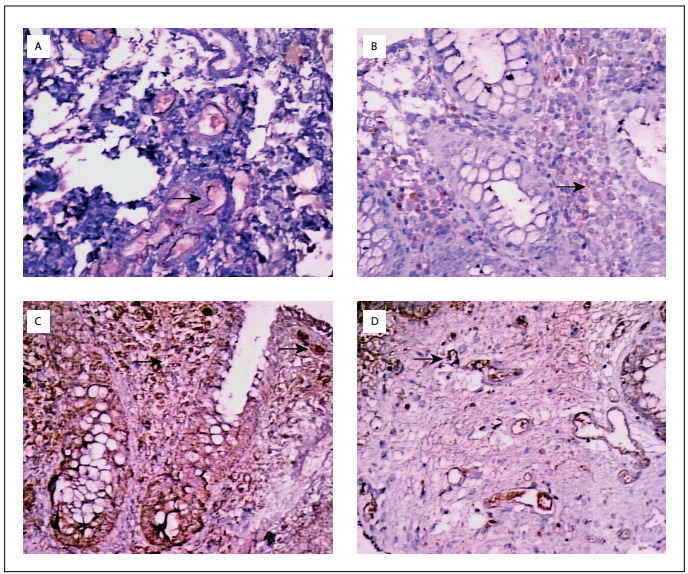
Immunohistochemical expression of vascular endothelial growth factor (VEGF) and endostatin in the colonic mucosa of ulcerative colitis patients. A: endothelial endostatin expression, x 400; B: stromal endostatin expression, x 400; C: stromal and epithelial VEGF expression, x 400; D: endothelial VEGF expression, x 400.

VEGF and endostatin expressions in the epithelium, stroma and vascular endothelium were evaluated. Intracytoplasmic staining was positive in the cells. The distribution of staining was categorized as follows: - no staining; + staining in a few cells; ++ staining approximately half of the cells; and +++ staining in the majority of cells. The intensity of staining was categorized as follows: + poor cytoplasmic staining; ++ significant cytoplasmic staining; and +++ severe cytoplasmic staining. The assessments were all made by the same pathologist, who was experienced in IBD. The H-scores were calculated by multiplying the overall stain distribution and intensity, in biopsies from the stroma, endothelium and epithelium separately (Figure 2).

### Statistical analysis

The Statistical Package for the Social Sciences (SPSS) for Windows, version 15.0 (SPSS Inc, Chicago, IL, USA), was used for statistical analysis. The results were given as the mean ± standard deviation or as the median with minimum-maximum. The normality of the distributions was checked.

To compare pairs of groups, the t test was used if the distribution was normal and the Mann-Whitney U test was used if the distribution was not normal. To make comparisons between more than two groups, analysis of variance (ANOVA) was used if the distribution was normal and the Kruskal-Wallis test was used if the distribution was not normal. The Mann-Whitney U test was used to compare pairs of subgroups. If the P value found in the test result was significant, multiple comparison tests were used to find out which group the difference originated from. P-values < 0.05 were considered statistically significant.

## RESULTS

There were no significant differences among the three groups in terms of age and gender. The demographic characteristics of the study population and the drugs used by the patients with UC during the serum sampling are presented in Tables 1and2. There was also no significant difference in immunohistochemical expression among the patients, in terms of age and gender. For UC patients (n = 33), the mean age was 45.4 ± 11.4 years and there were 13 females; while for IBS patients (n = 24), the mean age was 46.8 ± 12.1 years and there were 15 females.

**Table 1.** Baseline characteristics of the group with ulcerative colitis

Characteristic	n (%)
Disease duration, months (IQR)	72 (88)
Disease involvement site, n (%)	
Proctitis	7 (17.9)
Left colon site	16 (41)
Pancolitis	16 (41)
Disease activity, n (%)	
Mild	9 (23.1)
Moderate	22 (56.4)
Severe	8 (20.5)
Endoscopic activity, n (%)	
Inactive	20 (51.3)
Active	19 (48.7)
Types of drugs, n (%)	
ASA	32 (82.1)
Steroid	8 (20.5)
Metronidazole/ciprofloxacin/ampicillin	5 (12.8)
Azathioprine	5 (12.8)
Cyclosporine	1 (2.6)
None	5 (12.8)

IQR = interquartie range; ASA = aminosalicylic acid.

**Table 2.** Comparison of baseline characteristics of the study groups

	UC (n = 39)	IBS (n = 28)	HC (n = 15)	P-value	P-value: UC versus IBS	P-value: UC versus HC	P-value: IBS versus HC
Age, years	46.1 ± 12.6	48.2 ± 11.7	41.4 ± 12.6	0.232			
Female, n (%)	15 (38.5)	18 (64.5)	7 (46.7)	0.112			
VEGF (pg/ml)	511.9 ± 377.5	305.0 ± 121.4	236.1 ± 40.6	0.001*	0.007	≤ 0.001	0.709
VEGF, median (range)	420.8 (59.2-1700.2)	309.3 (108.6-638.1)	230.4 (190.5-340.4)	0.001**	0.032	0.019	0.044
Endostatin (ng/ml)	155.50 ± 59.8	116.9 ± 23.8	102.2 ± 22.4	< 0.001*	0.002	0.001	0.562
Endostatin, median (range)	156.1 (62.1-318.4)	115.3 (80.8-194.1)	98.5 (75.0-140.6)	0.001**	0.009	0.003	0.053

UC = ulcerative colitis; IBS = irritable bowel syndrome; HC = healthy control; VEGF = vascular endothelial growth factor; IQR = interquartile range; ASA = aminosalicylic acid. *This was studied using one-way analysis of variance. The homogeneity of the variances was tested by means of the Levene test. The post-hoc Tamhane test was used because the variances were not homogeneous. ** This was studied using the Kruskal-Wallis test. The Mann-Whitney U test was used in association with post-hoc analysis using the Bonferroni correction, and P < 0.017 was considered significant.

**Figure 3.**
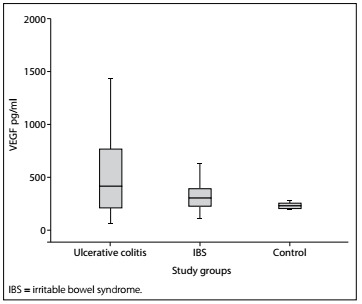
Serum vascular endothelial growth factor (VEGF) levels of the groups.

A statistically significant difference between the groups was found in terms of serum VEGF and serum endostatin levels (P = 0.001 and P < 0.001, respectively) (Table 1). The difference was due to the UC group. There was no statistically significant difference between the patients with IBS and the healthy controls in terms of serum VEGF and endostatin levels (P = 0.709 and P = 0.562, respectively) (Figures 3and4).

**Figure 4.**
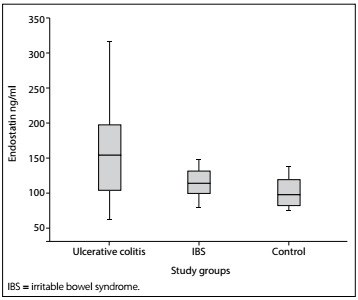
Serum endostatin levels of the groups.

Comparison between the mean serum VEGF and serum endostatin levels according to the clinical activity of the UC patients showed statistically significant differences (P = 0.004 and P = 0.011, respectively) (Table 3). There were significant differences between the groups with severe and moderate disease (P = 0.024) and between the groups with severe and mild disease (P = 0.04), but there was no significant difference between the groups with mild and moderate disease (P = 0.343), in terms of serum VEGF levels. There were statistically significant differences between the groups with severe and mild disease (P = 0.015) and between the groups with moderate and mild disease (P = 0.030), but there was no difference between the groups with severe and moderate disease (P = 0.625), in terms of serum endostatin levels.

**Table 3.** Serum vascular endothelial growth factor (VEGF) and endostatin levels in patients with ulcerative colitis (UC)

	Mild (n = 9)	Moderate (n = 22)	Severe (n = 8)	P-value	P-value: severe versus moderate	P-value: severe versus mild	P-value: moderate versus mild
VEGF (pg/ml)	290.3 ± 209.9	477.2 ± 283.7	856.8 ± 528	0.004*	0.024	0.04	0.343
VEGF, median (range)	229.9 (63.8-755.5)	425.0 (59.2-1037.3)	837.4 (75.4-1700.2)	0.018**	0.063	0.011	0.078
Endostatin (ng/ml)	107.2 ± 33.5	164.5 ± 60.0	185.3 ± 55.40	0.011*	0.625	0.015	0.030
Endostatin, median (range)	97.0 (74.9-182.2)	156.6 (62.1-318.4)	199.3 (75.7-271.0)	0.008**	0.344	0.011	0.004

*This was studied using one-way analysis of variance. The homogeneity of the variances was tested by means of the Levene test. The post-hoc Tukey test was used because the variances were homogeneous **This was studied using the Kruskal-Wallis test. The Mann-Whitney U test was used in association with post-hoc analysis using the Bonferroni correction, and P < 0.017 was considered significant.

Comparison between the mean serum VEGF and endostatin values according to the disease involvement site in patients with UC did not show any significant differences (P = 0.826 and P = 0.867, respectively). The mean serum VEGF level in the proctitis group was 430.0 ± 340.0 pg/ml; in the group with left colon involvement, 529.12 ± 375.1 pg/ml; and in the pancolitis group, 530.5 ± 412.7 pg/ml. The mean serum endostatin level in the proctitis group was 157.6 ± 54.1 ng/ml; in the group with left colon involvement, 160.7 ± 66.5 ng/ml; and in the pancolitis group, 149.4 ± 58.3 ng/ml.

Grouping of the patients with UC according to endoscopic activity index, i.e. as active (n = 19) or inactive (n = 20), showed significant differences in terms of mean serum VEGF levels (660.2 ± 416.5 pg/ml and 371.0 ± 278.9 pg/ml respectively; P = 0.011) and mean serum endostatin levels (179.8 ± 65.1 ng/ml and 132.4 ± 44.6 ng/ml respectively; P = 0.012)

There were statistically significant positive correlations between the serum VEGF and serum endostatin levels and the UCAI, ESR, CRP level and platelet count (Table 4).

**Table 4.** Spearman correlations for ulcerative colitis patients

	VEGF	Endostatin	ESR	CRP	Platelet	Albumin
UCAI	0.459**	0.475**	0.564***	0.564***	0.542***	-0.457**
VEGF		0.422**	0.703***	0.416**	0.542***	-0.401*
Endostatin			0.379*	0.398*	0.291	-0.272
ESR				0.727***	0.703***	-0.637***
CRP					0.669***	-0.429**
Platelet						-0.517***

UCAI = ulcerative colitis clinical activity index; VEGF = vascular endothelial growth factor; ESR = erythrocyte sedimentation rate; CRP = C-reactive protein. * P < 0.05; ** P < 0.01; *** irritable bowel syndrome, P < 0.001.

There were statistically significant differences in the stroma, endothelium and epithelium between the active UC, inactive UC and IBS groups in terms of H-scores of VEGF expression (P < 0.001). This difference was based on the active group (Table 5).

**Table 5.** H-scores for vascular endothelial growth factor (VEGF) and endostatin expressions in the mucosa of the active ulcerative colitis (UC), inactive UC and irritable bowel syndrome (IBS) groups

	Active UC (N = 33)	Inactive UC (N = 19)	IBS (N = 24)	P-value
H_VEGF_stroma	6.51 ± 3.49	2.38 ± 2.83	2.86 ± 1.58	< 0.001*
Median (range)	9 (0-9)	0 (0-9)	4 (0-4)	< 0.001*
H_VEGF_endothelium	5.26 ± 3.02	2.26 ± 2.92	2.50 ± 1.82	< 0.001*
Median (range)	6 (0-9)	0 (0-9)	2 (0-6)	< 0.001*
H_VEGF_epithelium	6.51 ± 3.34	2.64 ± 3.15	4.14 ± 2.99	< 0.001*
Median (range)	9 (0-9)	2 (0-9)	4 (0-9)	< 0.001*
H_endostatin_stroma	4.05 ± 3.05	1.87 ± 2.55	1.82 ± 1.52	< 0.001*
Median (range)	4 (0-9)	1 (0-9)	1 (0-4)	0.001*
H_endostatin_endothelium	2.08 ± 2.21	1.41 ± 2.12	0.86 ± 1.18	0.043**
Median (range)	1 (0-9)	0 (0-6)	0 (0-4)	0.059

*This difference was based on the active UC group (no difference between inactive and IBS). **There was a significant difference only between the active UC group and IBS group.

Endostatin expression was not viewed in the epithelium in any group. There were statistically significant differences in terms of H-score for endostatin expression in the stroma between the active UC, inactive UC and IBS groups (P < 0.001); whereas there was only a statistically significant difference in terms of H-score for endostatin expression in the endothelium between the active UC and IBS groups (P = 0.043).

## DISCUSSION

In the present study, we demonstrated for the first time that higher colonic mucosal expression of the potent angiogenic factor VEGF and the potent anti-angiogenic factor endostatin occurred, rather than elevated serum levels, in patients with UC. Comparison of serum VEGF and endostatin levels according to UCAI showed that the most notable difference was between the groups with severe and mild disease. In addition, there was a significant difference between the active and inactive groups, in terms of both serum VEGF and endostatin, through evaluation according to EAI. There was no difference between the groups through evaluation according to disease extent. There were positive correlations between UCAI and the VEGF, endostatin, ESR, CRP and platelet levels. Mucosal VEGF and endostatin expressions were higher in the active UC group than in the inactive UC group and IBS group. There was no difference in mucosal VEGF and endostatin expressions between the inactive UC group and the IBS group.

The role of angiogenesis in IBD is double-sided. While it is necessary for wound healing and tissue repair, it also promotes inflammation as a consequence of expansion of the tissue microvascular bed and the increased inflow and production of inflammatory cells and cytokines. In other words, angiogenesis contributes greatly towards the chronicity of inflammation.^20^

UC is a chronic inflammatory disease characterized by inflammation, ulceration and regeneration of the colonic mucosa.^1^ It has long been known that vascular changes occur in IBD. Abnormal vasculature, increased permeability and increased microvessel density have been reported previously. Danese et al. reported that the microvessel density was greater, along with the numbers of αVβ3-positive angiogenic vessels, in the mucosa of IBD patients. They indicated that the dense inflammation was due to local microvascular changes.^20^

Several proangiogenic molecules are involved in angiogenesis in IBD cases. VEGF is a potent angiogenic molecule. Several studies have now shown that serum and tissue VEGF concentrations become greater in IBD patients. It has also been shown that there is a correlation between disease activity and serum VEGF level in patients with IBD. Our results are similar to those from previous studies in terms of higher serum VEGF levels and higher mucosal VEGF expression, and a positive correlation of VEGF levels with disease activity.^5^^,^^6^^,^^7^^,^^9^^,^^10^^,^^11^

Angiogenesis is sustained by the balance between proangiogenic and antiangiogenic factors. Sandor et al. were the first to demonstrate the presence of increased expression of the antiangiogenic molecules endostatin and angiostatin instead of increased expression of VEGF, in an experimental model for colitis.^15^ They speculated that the antiangiogenic factors inhibited ulcer healing despite the presence of increased angiogenic factors and that this might be the reason for chronicity of colitis. Deng et al. demonstrated that mesalazine treatment reduced the expression of endostatin and angiostatin through restoring the balance between MMM-2 and MMM-9 via TNF-α inhibition.^16^ They did not demonstrate any effect of reducing the increase in VEGF expression.

Tolstonova et al. showed that there were concomitant increases in the levels of endostatin and VEGF.^21^ They found a correlation between colonic lesion size and endostatin and VEGF levels. Our study is compatible with their study in terms of the positive correlation between serum VEGF and endostatin levels. They stated that increased endostatin levels were a defensive response to the increased VEGF in UC. They also suggested that endostatin might be an alternative treatment for UC.

Konstatinos et al. found elevated levels of the proangiogenic molecules angiogenin and angiopoietin-2, along with higher serum levels of endostatin in UC patients with extensive colitis.^8^ We did not find any correlation between serum VEGF and endostatin levels and the extent of the disease. Salem et al. reported that the levels of endostatin, angiostatin, VEGF and TNF-α were reduced through niacin treatment.^22^

The complexity and range of pathways within the pathophysiology of chronic diseases have led to proposals for a number of options for treatments. The contribution of increased and unregulated angiogenesis in IBD, towards chronic inflammation, has long been known. It has been demonstrated that antiangiogenic therapy is effective against chronic inflammatory diseases such as arthritis, psoriasis and retinal neovascularization, and this suggests that it may also be effective for treating IBD.

The greatest limitations of our study were its cross-sectional nature and the small number of patients. Another limitation was that we were unable to evaluate mucosal expression in all of the patients whose serum levels of VEGF and endostatin we assessed. There is a need for prospective studies to show whether the serum or tissue endostatin level increase or decrease through treatment. Further experimental studies are also needed, to show the mucosal effect of local or systemic endostatin treatment.

## CONCLUSIONS

The serum and tissue VEGF and endostatin levels were found to be higher in patients with UC, and especially in those with active disease. This finding may explain the chronicity of the disease and may also form the basis of an idea for a new treatment option for UC patients.
